# Prevalence of multi drug resistant enteropathogenic and enteroinvasive *Escherichia coli* isolated from children with and without diarrhea in Northeast Indian population

**DOI:** 10.1186/s12941-017-0225-x

**Published:** 2017-07-11

**Authors:** Karuppasamy Chellapandi, Tapan Kumar Dutta, Indu Sharma, Surajit De Mandal, Nachimuthu Senthil Kumar, Lalsanglura Ralte

**Affiliations:** 1 Department of Veterinary Microbiology, Central Agricultural University, Selesih, Aizawl, Mizoram 796 014 India; 2 Department of Microbiology, Assam University, Silchar, Assam India; 3 Department of Biotechnology, Mizoram University, Aizawl, Mizoram India; 4 Department of MLT, Regional Institute of Paramedical and Nursing Sciences, Aizawl, Mizoram India

**Keywords:** EPEC, EIEC, Mizoram, Infants, MDR

## Abstract

**Background:**

Diarrheagenic *Escherichia coli* are associated with infantile diarrhea in the developing countries. The present study was conducted to determine the occurrence and antimicrobial resistance pattern of enteropathogenic and enteroinvasive *E. coli* associated with diarrhoea among the paediatric patients.

**Methods:**

A total of 262 stool samples were collected from children with and without diarrhea from Mizoram, Northeast India. *E. coli* were isolated and subjected to multiplex PCR to detect virulent genes of EPEC (*eaeA* and *bfpA*) and EIEC (*ial*). Isolates were subjected to antimicrobial sensitivity assay using disc diffusion method. Selected *eaeA* genes were sequenced for identification and genetic relationship.

**Results:**

A total of 334 *E. coli* was isolated, of which 17.37% were carrying at least one virulent gene. Altogether, 14.97 and 2.40% isolates were categorized as EPEC and EIEC, respectively. Among the DEC isolates, 4.79% were EPEC and 7.78% were EIEC. A total of 8 (2.40%) isolates were EIEC (*ial*+), of which 6 (1.80%) and 2 (0.60%) were from diarrhoeic and non-diarrhoeic patients, respectively. A total of 24 (41.40%) DEC isolates were MDR (resistance against ≥5 antimicrobials).

**Conclusions:**

A high frequency of EPEC pathotypes associated with paediatric diarrhea was observed in Mizoram, Northeast India and majority of the isolates are resistant to antibiotics with a high frequency of MDR, which is a matter of concern to the public health. This also raises an alarm to the world communities to monitor the resistance pattern and analyse in a global scale to combat the problems of resistance development.

## Background

Infectious diarrheal disease is the second leading cause of morbidity and mortality among children under 5 years of age in developing countries [1]. Diarrhea is common in Indian children with an incidence of 334,000 of total 2.3 million annual deaths [2]. Diarrheagenic *Escherichia coli,* specifically enteropathogenic *E. coli* (EPEC) is the leading bacterial agent causing diarrhoea in children aged below 5 years [3, 4]. EPEC isolates are divided into two groups: typical EPEC (tEPEC), which contains an EPEC adherence factor (EAF) for bundle forming pili (BFP) encoded by *bfpA* gene and atypical EPEC (aEPEC) is devoid of EAF [5]. For many decades tEPEC isolates were considered as the major pathogen in infants in developing countries including India, but only a few sporadic reports indicated the association of aEPEC with children diarrhoea [6–8].

Enteroinvasive *E. coli* (EIEC) is known to develop symptoms, which are similar to those of shigellosis in adults and children. Despite being recognized as a human pathogen, little research has been conducted to identify the risk factors for this infection so far. The lack of epidemiological attention to EIEC is related to the low incidence of this pathogen as a cause of diarrhea in relation to other strains of diarrheal *E. coli*. The genes related to invasion of EIEC are located in virulence plasmid (*p*Inv), which encodes a so-called type III secretion apparatus, the machinery required for the secretion of multiple proteins which are necessary for its full pathogenicity [9].

Apart from the conventional biochemical tests, the sensitive PCR based DNA assay helps in diagnosis of EIEC and detection of virulent genes like ipaH (Invasion plasmid antigen H) and *ial* (invasion-associated locus). The PCR based detection of *ial* gene for EIEC which is located on the pInv plasmid is a popular method, since it can also be effectively used to detect EIEC strains as well as other pathotypes of *E. coli* in a multiplex PCR [3, 10].

The emergence and spread of multi drug resistance (MDR) bacteria have been identified as a global burden in medical science and it stresses on surveillance monitoring to control the spread of MDR strains. Drug resistance in the developing countries has been attributed to the extensive uses of antibiotics and poor prescription practices. This problem may also affect the isolates recovered from children with diarrheal diseases. However, only a few studies have investigated the prevalence of diarrheagenic *E. coli* and their MDR patterns in India [8, 11, 12], where awareness and understating of MDR is still limited. Although sporadic reports on the association of EPEC and EIEC with children diarrhoea in India are available, the data is insufficient to understand the prevalence and strain variations to develop a National policy on management of the disease. The present study was formulated to investigate the prevalence of EPEC and EIEC from children with and without diarrhea in Northeast Indian Population. The other major aim of the study is to examine seasonal occurrence, multi drug resistance (MDR) pattern of the EPEC and EIEC isolates.

## Methods

### Clinical specimens and subjects

A total of 262 stool/rectal swab samples were collected from the childrens up to 5 years of age. Stool samples were collected using the sterile containers whereas rectal swab samples were collected using a sterile swab stick, preferably collected in the morning of the same day that the sample was processed. One sample from each patient was included in the present study and no repeated samples were collected from the same patient. Samples were collected between November 2013 to October 2015 from both inpatient and outpatient units of the major hospitals (Civil Hospital Aizawl, Aizawl Hospital & Research Centre and Presbyterian Hospitals Durtlang) of Mizoram, Northeast India. The patients were primarily of urban and suburban residents. Out of 262 samples, 210 samples were collected from children suffering from diarrhea with or without blood or mucus and the rest 52 samples were obtained from apparently healthy children but were admitted to the hospital for non-diarrheal illness. Clinical samples from the patients treated with antibiotics or infected with *Salmonella, Shigella* and co-infected with parasites and were not included in this study.

### Bacterial strains

All the samples were cultured on Mac Conkey’s agar (HiMedia Laboratories Pvt. Ltd., India) and incubated at 37 °C for 24 h. At least five randomly selected lactose fermenting pink coloured colonies were selected from each plate and were subjected to routine microbiological and biochemical tests to identify *E. coli* [13]. All the isolates were stored in glycerol at −80 °C for further studies.

### Serotyping

Serotyping of the *E. coli* isolates based on ‘O’ antigen was carried out at National Salmonella and Escherichia Centre, Central Research Institute (CRI), Himachal Pradesh, India as per the method of Edwards and Ewing [14].

### Preparation of bacterial DNA for PCR assay

Bacterial DNA was prepared using boiled lysis method. The isolates were inoculated into Luria–Bertani (LB) broth (HiMedia, Mumbai, India) and incubated overnight at 37 °C. The broth was centrifuged at 3000 rpm for 5 min. and the pellet obtained was dissolved in autoclaved distilled water followed by boiling for 10 min. The bacterial lysate was centrifuged again and the supernatant was finally taken as template DNA for PCR assay [15]. The specificity of the multiplex PCR assay was determined by using locally available and or reference strains as positive control strains (*E. coli* O20 serotype positive for *eaeA, bfpA* genes of EPEC and *E. coli* ATCC^®^ 43893 positive for *ial* gene of EIEC) for the standardization of multiplex PCR assay. The strains were subjected to both the multiplex and monoplex PCR assays. Both the multiplex and monoplex assays showed 100% specificity in identifying the reference strains and none of the reaction showed non-specific bands under UV-visualization.

### Detection of virulence genes by multiplex PCR

A multiplex PCR assay was carried out and the following specific primers were used: *eaeA* F (5′-TGATAAGCTGCAGTCGAATCC-3′), *eaeA* R (5′-CTGAACCAGATCGTAACGGC-3′), *bfp*A F (5′-CACCGTTACCGCAGGTGTGA-3′) *bfp*A R (5′-GTTGCCGCTTCAGCAGGAGT-3′) for the EPEC and *ial* F (5′-CTGGTAGGTATGGTGAGG-3′), *ial* R (5′-CCAGGCCAACAATTATTTCC-3′) for the EIEC as previously described [16]. The multiplex PCR reaction mixture contained 2.5 µl of 10× PCR buffer with 1.5 mM of MgCl_2_, 1 µl each primer, 2 µl of 10 mM each of dNTPs, 0.2 µl of 5.0 U Taq DNA polymerase and 4.0 µl template DNA. The PCR reaction condition includes initial denaturation at 95 °C for 5 min, followed by 32 cycles of denaturation at 94 °C for 45 s, annealing at 57 °C for 45 s and extension at 72 °C for 1 min, followed by a final extension at 72 °C for 5 min. Amplified products were separated on 1.5% agarose gel and stained with ethidium bromide and were visualized under ultraviolet transilluminator and documented by a gel documentation system (Alpha Imager, Germany).

### Cloning and sequence analysis

Selected PCR products were purified (QIAGEN kit) and cloned using TA cloning vector (MBI Fermentas) and sequenced in automated sequencer Applied Biosystems 3500 (USA) in the Dept. of Biotechnology, Mizoram University, India. Sequencing data were analysed using MEGA6 [17].

### Antimicrobial susceptibility test

The isolates positive for at least one virulence marker gene by PCR were subjected to antimicrobial susceptibility test against the selected antimicrobials (ampicillin-10 μg, amikacin-10 μg, chloramphenicol-30 μg, ceftriaxone-10 μg, cephalexin-30 μg, ciprofloxacin-10 μg, co-trimoxazole-25 μg, cefoperazone-tazobactam-75 + 10 μg, meropenem-10 μg, norfloxacin-10 μg, gentamicin-10 μg, cefixime-5 μg, doxycycline hydrochloride-10 μg and ofloxacin-5 μg) (HiMedia, India) by disc diffusion method in Mueller–Hinton agar [18]. The performance of this test was checked by employing *E. coli* ATCC 25922 as a standard quality control strain. The results were expressed as sensitive, intermediate and resistant as per standard CLSI guidelines [19]. MDR was defined as “acquired non-susceptibility to at least one agent in three or more antimicrobial categories” [20].

### Statistical analysis

Data were analysed using SPSS version 17.0 software. The significance (p < 0.05) of differences between the prevalence of EPEC and EIEC in age groups, Seasonal variations, the difference between MDR resistance occurrence and sample types were compared using a Chi square test for dependent samples or Fischer’s exact test when appropriate. The *p* values less than 0.05 was considered statistically significant.

## Results

### Isolation and identification of *E. coli* pathotypes

A total of 334 *E. coli* isolates were recovered from 262 faecal samples derived from 262 patients. Based on culture, staining, colony characteristics as well as biochemical tests, 282 and 52 *E. coli* were isolated from diarrhoeic and non-diarrhoeic patients, respectively.

### Clinical features of children affected with DEC

Occurrence of DEC among the female (10.3%) are little higher than male (11.8%) (p > 0.05 and 95% CI 0.456–9.86) though there is no role of sex factor as biological evidence, the mean age of the patients was 12 months (range from 2 to 36 months). Vomiting and abdominal pain were seen in 12.6 and 11.8% of children affected with EPEC and 1.5 and 1.9% in EIEC infected childrens, respectively. Childrens infected with EPEC (13%) and with EIEC (1.9%) showed fever at presentation (p > 0.05 and 95% CI 0.327–5.414). The consistency of the stool samples were mostly watery (68.7%), mucous (12.9%) and very few with blood (6.9%) with different number of episode per day and varied from mild to severe dehydration. Other clinical features with the pathotype are summarized in Table 1. The watery stool was found more common in EPEC infection (15.3%) than any other consistency such as mucous (1.1%) and blood (0.3%) with mild dehydration (4.96%) and only a few cases show severe dehydration (1.5%). When compared with EPEC infections, EIEC cases have shown more mucous stools (0.76%) and bloody stool (1.5%) than watery stools (0.3%) with mild dehydration (0.3%) only (p < 0.005 and 95% CI 5.70–631.59).

**Table 1:** Clinical symptoms of subjects included in this study

Clinical characteristics	Total no. of subjects (n = 262)	EPEC (n = 50)	EIEC (n = 08)
Sex ratio			
Male	114 (43.5)	28 (10.69)	03 (1.14)
Female	148 (56.5)	22 (08.39)	05 (01.91)
Fever			
Febrile	123 (46.9)	34 (13.00)	05 (01.91)
Afebrile	139 (53.0)	13 (04.96)	02 (0.76)
Vomiting	91 (34.7)	33 (12.60)	04 (01.52)
Abdominal pain	48 (18.3)	31 (11.80)	05 (01.91)
Diarrhea and non-diarrhea			
Watery stool	158 (60.3)	40 (15.30)	01 (0.38)
Mucous	34 (12.9)	03 (01.15)	02 (0.76)
Bloody	18 (06.9)	01 (0.38)	04 (01.52)
No symptoms	52 (19.84)	03 (01.15)	–
Diarrheal frequency			
2–3 times a day	180 (68.7)	27 (10.31)	07 (02.67)
4–5 times a day	19 (07.25)	19 (07.25)	–
5–10 times a day	11 (04.2)	01 (0.38)	–
Dehydration			
Mild dehydration	37 (14.1)	13 (04.96)	01 (0.38)
Moderate dehydration	28 (10.7)	14 (05.34)	–
Severe dehydration	63 (24.0)	04 (01.53)	–
Unknown	134 (51.1)	16 (06.11)	06 (02.30)

Within parenthesis denotes the % of the samples

*EPEC* enteropathogenic *E. coli*, *EIEC* enteroinvasive *E. coli*

### Distribution of DEC

As depicted in Table 2, of the total 334 *E. coli* isolates subjected for detection of EPEC (*eaeA*) and EIEC (*ial*) marker genes, 58 (17.36%) were positive for at least one of the target gene, of which 50 (14.97%) and 8 (2.33%) isolates were categorized as EPEC and EIEC, respectively. Among the 8 EIEC isolates, 6 (1.80%) and 2 (0.59%) were recovered from diarrhoeic and non-diarrhoeic patients, respectively. Similarly, amongst the EPEC isolates, 38 (11.38%) and 12 (3.59%) were isolated from the diarrhoeic and non-diarrhoeic patients, respectively (p > 0.05 and 95% CI 0.187–5.938). Among the EPEC isolates, 21 (6.29%) were also positive for *bfp*A gene, hence termed as typical EPEC (tEPEC) isolates and rest 29 (8.68%) were categorized as aEPEC. Among the aEPEC isolates, 22 (6.59%) were isolated from diarrhoeic patients and 7 (2.10%) were recovered from non-diarrhoeic patients. Although the majority of the tEPEC were isolated from diarrhoeic patients, 5 (1.50%) isolates were also recovered from non-diarrhoeic patients (p > 0.05 and 95% CI 0.273–3.797). In case of EIEC, 2 (0.59%) isolates were recovered from children without diarrhea.

**Table 2:** Frequency of *E. coli* pathotypes isolated from fecal samples of children with and without diarrhea

S. no	*E. coli* pathotypes	*E. coli* isolates from non-diarrheic patients	*E. coli* isolates from diarrheic patients	Total (n = 334)
1.	EPEC	12 (3.59)	38 (11.38)	50 (14.97)
	*a*-*EPEC*	07 (2.10)	22 (6.59)	29 (8.68)
	*t*-*EPEC*	05 (1.50)	16 (4.79)	21 (6.29)
2.	EIEC	02 (0.59)	06 (1.80)	08 (2.33)
Total		14 (4.07)	44 (13.17)	58 (17.37)

Figures in parenthesis denotes the % of the samples

*a-EPEC* atypical enteropathogenic *E. coli, t-EPEC* typical enteropathogenic *E. coli*

The incidence of acute diarrhea associated with EPEC and EIEC decreased significantly with the increasing age of the patient, and was highest (6.6%) in children aged 13–24 months and lowest among the age group of 37–48 months, which is significantly low (0.6%) compared with other age groups (Table 3) (p > 0.05 and 95% CI 0.043–28.547). Incidence in the age group of 0–12 months was lower (6.3%) than the weaning age group of children in Mizoram. The occurrence of aEPEC in children aged with 0–24 months (5.3%) was more than other age groups, and it’s also found as not associated with diarrhea in 0–12 months children (0.9%) of the non-diarrheic group. Rate of tEPEC occurrence in the age group of 0–24 months (1.2%) is higher when compared with other age groups; occurrence in non-diarrheic cases shows the high prevalence of tEPEC carriers. All the EIEC pathotypes were recovered from the age group of 0–24 months except one isolate found in 49–60 group. Though the occurrence of EIEC in non-diarrheic group is meager (0.3%) its occurrence in non-diarrheic cases causes a confused state of the pathogenicity of this pathotype. Incidence of diarrhea associated with EPEC/EIEC among the young infants and children of Mizoram was highest (8.08%) during summer followed by monsoon (5.09%) (p > 0.05 and 95% CI 0.275–2.589) and winter (4.20%) (Table 4).

**Table 3:** Age wise comparison of EPEC and EIEC from the stool samples of children

Age (months)	No. of isolates	Atypical EPEC (n = 29)		Typical EPEC (n = 21)		EIEC (n = 08)		Total (n = 334)
		Age	Non age	Age	Non age	Age	Non age	
0–12	176 (52.69)	07 (02.09)	03 (0.90)	06 (01.80)	01 (0.30)	03 (0.90)	01 (0.30)	21 (6.3)
13–24	98 (29.34)	11 (03.29)	–	05 (01.50)	03 (0.90)	02 (0.60)	01 (0.30)	22 (6.6)
25–36	37 (11.07)	05 (01.49)	–	01 (0.30)	01 (0.30)	–	–	7 (2.1)
37–48	14 (04.19)	01 (0.30)	–	01 (0.30)	–	–	–	2 (0.6)
49–60	09 (02.69)	02 (0.60)	–	03 (0.90)	–	01 (0.30)	–	6 (1.8)
Total	334	26 (7.8)	03 (0.9)	16 (4.8)	05 (1.5)	06 (1.8)	02 (0.5)	58 (17.4)

Figures in parenthesis denotes the % of the samples

*AGE* acute gastroenteritis, *EPEC* enteropathogenic *E. coli*, *EIEC* enteroinvasive *E. coli*

**Table 4:** Seasonal distribution of diarrheagenic *E. coli* among children

Seasons	*E. coli* isolates	‘O’ Serotypes (no. of isolates)	EPEC		EIEC (%)	Total (DEC) (%)
			Atypical (%)	Typical (%)		
Summer	138	EPEC (22): O2 (1), O26 (2), O84 (1), O86 (2), O91 (1), O116 (1), O 129 (1), O141 (4), O145 (1), O149 (3), Rough (2), UT (3)	12 (3.59)	10 (2.99)	05 (1.5)	27 (8.08)
		EIEC (05): O29 (1), O96 (2), O124 (1) UT (1)				
Rainy/autumn	96	EPEC (16): O2 (1), O 29 (1), O 83 (1), O84 (3), O86 (2), O91 (1), O126 (1), Rough (4), UT (2)	10 (2.99)	06 (1.8)	01 (0.3)	17 (5.09)
		EIEC (01): UT (01)				
Winter	100	EPEC (12): O 2 (1), O 35 (1), O83 (1), O 101 (1), O126 (2), O 145 (2), O 149 (1), Rough (1), UT (2)	07 (2.1)	05 (1.5)	02 (0.6)	14 (4.19)
		EIEC (02): O20 (1), O144 (1)				
Total	334	58	29 (8.68)	21 (6.29)	08 (2.4)	58 (17.37)

Figures in parenthesis denotes the % of the samples

*EPEC* enteropathogenic *E. coli, EIEC* enteroinvasive *E. coli*

### Serotypes

The O serotypes detected in this study from diarrheagenic *E. coli* isolates were corroborating with the previously reported serotypes and association with childhood diarrhea with few novel serotypes. The majority of the isolates of typical and atypical EPEC belonged to the serogroups: O 84 (4), O 86 (4), O141 (4), O149 (4), Rough (7) and UT (7). All the isolates found to be EIEC belonged to O serogroups of O20 (1), O29 (1), O96 (2), O124 (1) O144 (1) and UT (2). The occurrence of these O serogroups in three different seasons was also studied to understand their occurrences and association with childhood diarrhea (Table 4).

### Antimicrobial resistance of *E. coli* isolates

Antibiotics commonly used by local medical practitioners for the treatment of childhood diarrhea were included in this study for the antibiotic susceptibility tests. None of the diarrheagenic *E. coli* isolates were sensitive against all the antimicrobials used in this study. Altogether, 41.40% of DEC was recorded as multi drug resistant (MDR) of which >70% of aEPEC and 50% of tEPEC isolates were resistant to ampicillin, ceftriaxone, cefoperazone-tazobactam, cefixime and doxycycline. Similarly, the EIEC isolates also exhibited high rate (60–80%) of resistance to majority of the antibiotics used in the study (Table 5). The MDR isolates against the cephalosporin drugs (83%) still higher than other drugs including aminoglycosides, carbapenem, fluoroquinolone and sulphonamides (16.9%) used in this study (p > 0.05 and 95% CI 0.830–3.008).

**Table 5:** Antimicrobial resistance profile of diarrheagenic *E. coli* isolated from stool samples collected from children

DEC (n)	AMP	AK	C	CN	CI	CF	COT	CST	MRP	NX	G	CFM	DO	OF	MDR (%)
a-EPEC (29)	21 (72.4)	06 (20.7)	14 (48.3)	14 (48.3)	22 (75.9)	10 (34.5)	09 (31.0)	24 (82.8)	06 (20.7)	12 (41.4)	04 (13.8)	21 (72.4)	12 (41.4)	14 (48.3)	14 (48.3)
t-EPEC (21)	14 (66.7)	08 (38.1)	05 (23.8)	12 (57.1)	12 (57.1)	03 (14.3)	08 (38.1)	16 (76.2)	06 (28.6)	09 (42.6)	07 (33.3)	12 (57.1)	12 (57.1)	07 (33.3)	07 (24.1)
EIEC (08)	07 (87.5)	01 (12.5)	00 (0)	06 (75)	07 (87.5)	04 (50)	07 (87.5)	04 (50)	05 (62.5)	01 (12.5)	05 (62.5)	05 (62.5)	05 (62.5)	05 (62.5)	03 (37.5)
Total (58)	42 (72.4)	15 (25.9)	19 (32.8)	32 (55.2)	41 (70.7)	17 (29.3)	24 (41.4)	44 (75.9)	17 (29.3)	22 (37.9)	16 (27.6)	38 (65.5)	29 (50)	26 (44.8)	24 (41.4)

Figures in parenthesis denotes the % of the samples

*MDR* multi drug resistant, *a-EPEC* atypical enteropathogenic *Escherichia coli*, *t-EPEC* typical enteropathogenic *Escherichia coli*, *EIEC* enteroinvasive *Escherichia coli*, *μg* microgram, *Antibiotics* ampicillin (AMP10 = 10 μg), amikacin (AK10 = 10 μg), chloramphenicol (C 30 = 30 μg), ceftriaxone (CI 10 = 10 μg), cephalexin (CN30 = 30 μg), ciprofloxacin (CF10 = 10 μg), co-trimoxazole (COT25 = trimethoprim 1.25 μg and sulphamethoxazole 23.75 μg), cefoperazone–tazobactam (CST 75–10 μg), meropenem (MRP10 = 10 μg), norfloxacin (NX10 = 10 μg), gentamicin (G10 = 10 μg), cefixime (CFM 5 = 5 μg), doxycycline hydrochloride (DO10 = 10 μg) and ofloxacin (OF 5 = 5 μg)

## Discussion

*Escherichia coli* is identified as an important cause of paediatric diarrhea in developing countries. Although DEC pathotypes are well recognized, they are not routinely sought due to lack of infrastructures such as antisera and advanced molecular techniques. Thus, the exact burden of *E. coli* diarrhea among the hospitalized children across India especially Northeast India is still unclear. In the present study, the samples were collected from Mizoram, located in the North Eastern part of India adjoining to Myanmar and Bangladesh where limited study has been conducted on the prevalence of diarrhea associated with DEC [15, 21]. This is the first report on an in-depth study on the prevalence of EPEC and EIEC associated with diarrhea in children less than 5 years of age in North-eastern Indian population. In the present study, children with DEC diarrhea were characterized by the presence of fever, profuse watery stools, more than 3–5 stools per 24 h, associated with vomiting and mild to severe dehydration as common clinical features. The occurrence of EPEC was much higher compared to EIEC and both the pathotypes were higher in febrile patients than afebrile patients. Similar clinical symptoms were observed in patients from other parts in India [6, 8].

Among all the DEC, EPEC found to be the most common pathotypes for diarrheal infection in Mizoram which is much higher than the previous report [15]. Recent studies from other part of India also found higher occurrence of EPEC compared to EIEC among diarrheal samples [8, 16, 22]. Of the 50 EPEC isolates from the present study, 11.38% were recovered from the diarrhoeic patients in comparison with the non-diarrhoeic patients 3.59%, which is probably good evidence for their role in development of diarrhea among the children. But at the same time presence of EPEC isolates from the population of the non-diarrhoeic patients also indicates their role as potent carrier of the organism, which may infect the other vulnerable population of the locality. In non-diarrheic group, both atypical 2.1% and typical 1.5% EPEC were detected which is very less than the previous reports in India [7, 16]. For many decades tEPEC isolates were considered as the major pathogen in infants in developing countries including India [6, 22], but few sporadic reports indicated the association with aEPEC with children diarrhea [6, 7, 22]. In contrast to typical EPEC, atypical EPEC strains appear to be common in domestic animals, raw meats and have been reported in food borne and water borne outbreaks [5, 9, 15]. This might be the possible source of transmission of this pathogen among children through mother, baby sitter or other members of the family.

In India, very few reports are available the occurrence of EIEC associated with children diarrhea. In the present study, a total of 2.33% *E. coli* isolates were confirmed as EIEC, of which 0.59 and 1.80% were from non-diarrhoeic and diarrhoeic patients, respectively. Other studies in India also reported less percentage of EIEC (1–1.5%) only in diarrheal patients [8, 16, 22]. The presence of EIEC strains in non-diarrheic group suggests that their prevalence is high in this study region among other age groups or sources including contaminated food and water [9]. Detection of EIEC in association with infants or children is a paramount important issue for effective treatment and control of the disease. Given the potential importance of this invasive pathogen, more work should be focused on why EIEC is prevalent in children of non-diarrheic group of this study region.

Considering the age groups, the EPEC ad EIEC strains were detected in almost all the age groups, but significantly higher prevalence was seen in children over 12 months of age (p < 0.05). Almost similar rate of occurrence was seen in 0–12 month age group, where the children’s are completely dependent on mother. Thus, a proper hygienic condition of mother prior to breastfeeding could significantly reduce mother-to-infant transmission of this DEC. Of the 2.3% EIEC strain all of them occurred in the age group of 0–24 month except one single strain detected in the age group of 49–60 month. Occurrence of these age groups children as a pathogen indicates there might be some immune related issues in the particular individual as there are studies reported that these pathogens infecting the children enhance immunity as they grow into adults and then they act as carriers of this pathogen [9]. Seasonal distribution of the DEC in Aizawl, Mizoram showed that most of the cases occurred in summer probably owing to the increase in temperature, followed by rainy and winter seasons. Similar results were found in other parts of India and outside India [11, 23, 24]. Serotype associated with a particular pathotypes was similar with the previous studies [7, 9]. Occurrence of serotypes in different seasons was also considered in this study and no seasonal variation was observed.

Many factors play a role related to the high prevalence of diarrhea, including lack of education of mother, lack of exclusive breastfeeding, breastfeeding for less than 1 year, roundworm infestation, nutritional status, immunization status, night blindness, personal hygiene, overcrowding, garbage disposal, source of water supply and toilet facility. Earlier studies has reported that occurrence of diarrhea is higher (31.57%) in children who were breastfed for less than 6 months compared to exclusively breastfed children (20.33%) and bottle-fed children (26.08%). It was found that mixed-fed infants aged between 0 and 11 months tend to have a higher risk of diarrhea than fully breastfed children [25].

As per WHO protocol, paediatric diarrheal patients should be treated with rehydration solution, intravenous fluids, oral zinc suspensions, but failure of this protocol lead to MDR in bacterial pathogens [26]. High prevalence of MDR-EPEC strains was recorded in different parts of the world [27–29]. In the present study, antimicrobial resistance pattern was performed for all the DEC. Both tEPEC and aEPEC showed high levels of resistance to almost all the generic drugs used in this study such as aminoglycosides, cephalosporin, fluoroquinolone and tetracycline, but a very low level of resistance to carbapenem drugs. And the EIEC isolates were not only resistant to the commonly used genera drugs, but also to the fluoroquinolone and carbapenem. The aEPEC showed a significantly higher rate of resistance to antimicrobials than tEPEC against the selective antibiotics. It may be due to the fact that aEPEC was circulating in the human environment for a longer period in this region than the tEPEC, which got lesser exposure to the commonly used antimicrobials. In contrast to our result Sunaifa et al. [12] reported the higher antimicrobial resistance to the tEPEC than aEPEC from paediatric patients. The rate of adaptive mutation in *E. coli* is high which might be a cause of emergence of resistant varieties of aEPEC [6, 30]. The result of the present study indicates that aEPEC is the major DEC in Mizoram and also showing higher level of resistance against antimicrobials. The EIEC isolates recovered from this region showed high level of resistance against commonly used antimicrobials including fluoroquinolone and carbapenem, which is a clear indication of indiscriminate use of antimicrobials in clinical practice and sale across the counter in this region.

This is the first report on drug resistance EIEC isolated from Children in India. The emergence of widespread resistance to quinolone and other cephalosporin antimicrobials like cefixime, cefoperazone, and cephalexin etc. among diarrheal isolates is an important concern in this region. But the resistance rates against some of the broad spectrum antibiotics like gentamicin, amikacin, ciprofloxacin and meropenem were limited and can be used for the treatment of MDR diarrheal pathogens.

Therefore, it may be concluded that antibiotic resistant DEC is highly prevalent among the young population of Mizoram, India. Atypical EPEC are more prevalent compared to the typical EPEC and the aEPEC showed high level of resistance against the commonly used antimicrobial agents. High level of resistance development towards common antibiotics as well as quinolone and carbapenem in the EIEC isolates from human patients from North Eastern Region of India has been reported for the first time in the study. This study highlights the need for proper microbial diagnosis before antibiotic treatment, especially in the developing and under-developed regions of the world, where antibiotic abuse is widespread and a major growing concern.

## References

[1] Bryce J, Boschi-Pinto C, Shibuya K, Black RE, WHO Child Health Epidemiology Reference Group (2005). WHO estimates of the causes of death in children. Lancet.

[2] Bassani DG, Kumar R, Aswathi S, Morris SK, Paul VK, Shet A, Ram U, Gaffey MF, Black RE, Jha P, Million death study collaborators (2010). Causes of neonatal and child mortality in India: a nationality representative mortality survey. Lancet.

[3] Nataro JP, Kaper JB (1998). Diarrheagenic *Escherichia coli*. Clin Microbiol Rev.

[4] Clarke SC, Haigh RD, Freestone PPE, Williams PH (2003). Virulence of enteropathogenic *Escherichia coli*, a global pathogen. Clin Microbiol Rev.

[5] Yanmei X, Xiangning B, Ailan Z, Wang Z, Pengbin B, Kai L, Yujuan J, Hong W, Quisheng G, Hui S, Jianguo X, Yanwen X (2016). Genetic diversity of intimin gene of atypical enteropathogenic *Escherichia coli* isolated from human, animals and raw meats in China. PLoS ONE.

[6] Ghosh PK, Ali A (2010). Isolation of atypical enteropathogenic *Escherichia coli* from children with and without diarrhea in Delhi and the National Capital Region, India. J Med Microbiol.

[7] Wani SA, Asifa N, Iffiat F, Irfan A, Yoshikazu N, Khursheed Q, Khan MA, Chowdhary J (2006). Investigation of diarrheic faecal samples for enterotoxigenic, Shiga toxin-producing and typical or atypical enteropathogenic *Escherichia coli* in Kashmir, India. FEMS Microbiol Lett.

[8] Rajeshwari K, Beena U, Singh R, Tiwari G, Ajay KM (2015). Multi drug resistant enteropathogenic *E. coli* diarrhea in children. Am J Res Commun.

[9] Vieira N, Bates SJ, Solberg OD, Ponce K, Howsmon R, Cevallos W, Trueba G, Riley L, Eisenberg JN (2007). High prevalence of enteroinvasive *Escherichia coli* isolated in a remote region of Northern Costal Ecuador. Am J Trop Med Hyg.

[10] Lanjewar M, Anuradha SD, Mathur M (2010). Diarrheagenic *E. coli* in hospitalized patients: special reference to Shiga-like toxin producing *Escherichia coli*. Indian J Pathol Microbiol.

[11] Sudershan RV, Kumar RN, Bharathi K, Kashinath L, Bhaskar V, Polasa K (2014). *E. coli* pathotypes and their antibiotic resistance in young children with diarrhea in Hyderabad, India. Int J Curr Microbiol App Sci.

[12] Sunaifa SM, Roy S, Dhanashree B (2015). Prevalence of enteropathogenic *Escherichia coli* (EPEC) in adult diarrhoea cases and their antibiotic susceptibility pattern. Br Microbiol Res J.

[13] Collee JG, Miles RS, Wan B, Collee JG, Fraser AG, Marmion BP, Simmons A (1996). Tests for the identification of bacteria. Mackie and McCartney practical medical microbiology.

[14] Edwards PR, Ewing WH (1986). Identification of Enterobacteriaceae.

[15] Begum J, Dutta TK, Chandra R, Chodhury PR, Varte Z, Bitew M (2014). Molecular and phenotypic characterization of shiga toxigenic *Escherichia coli* (STEC) and enteropathogenic *E. coli* from piglets and infants associated with diarrhoea in Mizoram, India. Afr J Biotechnol.

[16] Hegde A, Ballal M, Shenoy S (2012). Detection of diarrheagenic *Escherichia coli* by multiplex PCR. Indian J Med Microbiol.

[17] Tamura K, Stecher G, Peterson D, Filipski A, Kumar S (2013). MEGA6: molecular evolutionary genetics analysis version 6.0. Mol Biol Evol.

[18] Bauer AW, Kirby MD, Sherries JC, Truck M (1966). Antibiotic susceptibility testing by a standardized single disk method. Am J Clin Pathol.

[19] Clinical Laboratory Standards Institute. M100-S22 (2012). Clinical and Laboratory Standards Institute: performance standards for antimicrobial susceptibility testing. 22nd informational supplement, vol. 31, no. 1. (M100-S22). Villanova: Clinical and Laboratory Standards Institute; 2012.

[20] Magiorakos AP, Srinivasan A, Carey RB, Carmeli Y, Falagas ME, Giske CG (2012). Multidrug-resistant, extensively drug-resistant and pandrug-resistant bacteria: an international expert proposal for interim standard definitions for acquired resistance. Clin Microbiol Infect.

[21] Dutta TK, Warjri L, Roychoudhury P, Lalzampuia H, Samanta I, Joardar SN, Bandyopadhyay S, Chandra R (2013). Extended-spectrum β-lactamase producing *Escherichia coli* isolate possessing the shiga toxin gene (stx1) belonging to the O 64 serogroup associated with human diseases in India. J Clin Microbiol.

[22] Rajendran P, Ajjampur SS, Chidambaram D, Chandrabose G, Thangaraj B, Sarkar R, Samuel P, Rajan DP, Kang G (2010). Pathotypes of diarrheagenic *Escherichia coli* in children attending a tertiary care hospital in south India. Diagn Microbiol Infect Dis.

[23] Jafari F, Shokrzadeh L, Hamidian M, Salmanzadeh AS, Zali MR (2008). Acute diarrhea due to enteropathogenic bacteria in patients at Hospitals in Iran. Jpn J Infect Dis.

[24] Dallal MMS, Khorramizadeh MR, Ardalan KM (2006). Occurrence of enteropathogenic bacteria in children under 5 years with diarrhea in south Tehran. East Mediterr Health J.

[25] Gupta A, Gautam S, Arup JR, Tanushree M, Ranabir P (2015). Risk correlates of diarrhea in children under 5 years of age in slums of Bankura, West Bengal. J Glob Infect Dis.

[26] World Health Organization. Treatment of diarrhea—a manual for physicians and other senior health workers, WHO/CAH/03.1. Geneva: World Health Organization. http://whqlibdoc.who.int/publications/2005/9241593180.pdf.

[27] Scaletsky ICA, Souza TB, Aranda KRS, Okeke IN (2010). Genetic elements associated with antimicrobial resistance in enteropathogenic *Escherichia coli* (EPEC) from Brazil. BMC Microbiol.

[28] Dhanashree B, Mallya PS (2012). Molecular typing of enteropathogenic *Escherichia coli* from diarrheagenic stool samples. J Clin Diagn Res.

[29] Farthing M, Salam MA, Lindberg G, Dite P, Khalif I, Salazar-Lindo E, Krabshuis J (2013). Acute diarrhea in adults and children: a global perspective. J Clin Gastroenterol.

[30] Perfeito L, Fernandes L, Mota C, Gordo I (2007). Adaptive mutations in bacteria: high rate and small effects. Science.

